# Independent Journalism

**Published:** 1880-05

**Authors:** 


INDEPENDENT MEDICAL JOURNALISM.—We refer to this subject again, to pay a passing tribute to one of the best and truest members of the medical editorial corps of this country — to a gentleman of enlightened sagacity and magnanimous intrepidity in the discharge of the duty he owes to a profession more grievously encumbered just now and unfairly used in many vitally important respects than any other calling whatsoever. This gentleman is exerting a grand influence through the editorial columns of the *Medical Record*, in his fearless attacks upon the "irregularities," "medical charities," "appointment of public officials," etc., etc., and is therefore entitled to the esteem of those who desire to interpose for the rescue of a noble calling from impending degradation and disgrace. Much to be admired and applauded, too, should he be who wields a wise and fearless pen in upholding and defending the most ancient, as it was at one time the most honorable and important callings to humanity, and one, too, that has often received the endorsement and culture of the wise and good among men. Perhaps there never has been a period in the history of the healing art that called as urgently for the exercise of loyalty to the banner of Æsculapius, and that required each one in the editorial ranks "to do his whole duty" fearlessly and uncompromisingly, than the present. Error holds up its brazen face, and apostasy and dissimulation wear disguises of so flimsy texture that the inexperienced eye can see the deformities heretofore more deftly and artistically concealed. The dominant idea seems to be now in the race for medical work, that "he may take who has the power and he may keep who can." The world admires and applauds its heroes, philosophers and poets, but those who shall manfully fight this great battle for the rights of our common humanity will be entitled to, and will doubtless "receive the plaudit of well done" in the ages yet to come, if not now. We are pleased to add, ere we conclude, that while allusion is directly made to the able editor of the *Record* in terms of merited commendation, there are others entitled to our meed of praise in no stinted measure, and it will be our pleasure to refer to them from time to time as the work of cleansing the medical "stables" advances. It has been said that the most effective preachers are those who have had personal experiences among sinners and with the interior condition of things of which they may speak. But whether the same rule will apply to the medical editor with much force or not, there are some transactions we have known of in the past that are recognized as largely contributing factors in the terribly demoralized condition of things now being ventilated by the more enlightened and independent portion of the medical press; and it is our purpose to "skirmish" whilst the heavier artillery are moving "on this line," until existing evils are suppressed or overthrown entirely.

